# Searching for rare diseases in PubMed: a blind comparison of Orphanet expert query and query based on terminological knowledge

**DOI:** 10.1186/s12911-016-0333-0

**Published:** 2016-08-02

**Authors:** N. Griffon, M. Schuers, F. Dhombres, T. Merabti, G. Kerdelhué, L. Rollin, S. J. Darmoni

**Affiliations:** 1 Department of Biomedical Informatics, Rouen University Hospital, TIBS, LITIS EA 4108, Rouen University, 76031 Rouen Cedex, France; 2 INSERM, U1142, LIMICS, 75006, Paris, France; Sorbonne Universités, UPMC Univ Paris 06 UMR_S 1142, LIMICS, 75006, Paris, France; Univ Paris 13, Sorbonne Paris Cité, LIMICS (UMR_S 1142), 93430 Villetaneuse, France; 3 Department of Family Practice, Rouen University, Rouen, France; 4 Service de Médecine Fœtale, Hôpital Trousseau – Hôpitaux Universitaires de l’Est Parisien (APHP), Université Pierre et Marie Curie, Paris, France; 5 Department of Occupational Medicine, Rouen University Hospital, Rouen, France

**Keywords:** PubMed, Rare diseases, Bibliography as topic, Terminology as topic

## Abstract

**Background:**

Despite international initiatives like Orphanet, it remains difficult to find up-to-date information about rare diseases. The aim of this study is to propose an exhaustive set of queries for PubMed based on terminological knowledge and to evaluate it versus the queries based on expertise provided by the most frequently used resource in Europe: Orphanet.

**Methods:**

Four rare disease terminologies (MeSH, OMIM, HPO and HRDO) were manually mapped to each other permitting the automatic creation of expended terminological queries for rare diseases. For 30 rare diseases, 30 citations retrieved by Orphanet expert query and/or query based on terminological knowledge were assessed for relevance by two independent reviewers unaware of the query’s origin. An adjudication procedure was used to resolve any discrepancy. Precision, relative recall and F-measure were all computed.

**Results:**

For each Orphanet rare disease (*n* = 8982), there was a corresponding terminological query, in contrast with only 2284 queries provided by Orphanet. Only 553 citations were evaluated due to queries with 0 or only a few hits. There were no significant differences between the Orpha query and terminological query in terms of precision, respectively 0.61 vs 0.52 (*p* = 0.13). Nevertheless, terminological queries retrieved more citations more often than Orpha queries (0.57 vs. 0.33; *p* = 0.01). Interestingly, Orpha queries seemed to retrieve older citations than terminological queries (*p* < 0.0001).

**Conclusion:**

The terminological queries proposed in this study are now currently available for all rare diseases. They may be a useful tool for both precision or recall oriented literature search.

**Electronic supplementary material:**

The online version of this article (doi:10.1186/s12911-016-0333-0) contains supplementary material, which is available to authorized users.

## Background

There is currently no consensual definition of what is a rare disease: in Europe, a disease is considered rare if it affects less than 1 in 2000 citizens, while in United States of America (USA), the threshold was set at 200,000 in the entire population [1] (approximately 1 in 1600 according to the USA census bureau [2]).

These gross definitions lead to a major heterogeneity between rare diseases:

- Most of genetic diseases are rare diseases, but some infectious diseases, cancer and auto-immune diseases are also rare.
- They may occur at any point in life
- There are geographical variations. A disease may be rare in one country (like Periodic disease in France) but quite frequent in another (Periodic disease in Armenia)
- Some are well known and have been described for a number of years, whereas some have been recently discovered and information is scarce.

Furthermore, these definitions have led to the knowledge of 5000 to 8000 rare diseases and to the “paradox of rarity”: each disease is rare, but patients with rare diseases are numerous. Having a clear vision of the prevalence of rare diseases is not an easy task, nevertheless, it is commonly accepted that approximately 5 to 10 % of the population suffer from rare diseases (8–9 % in the USA [1], 6–8 % in the European Union [3]). In both regions, this corresponds to approximately 30,000,000 patients suffering from a rare disease, making it a real public health concern [4].

This heterogeneity and frequency of rare diseases translates into numerous different situations in which some information is needed:

- Finding a physician with adequate experience may be easy when a reference center exists, but can be a real difficulty if care pathways are not identified [5]
- Providing medical care for patients with a rare disease is a difficult task for physicians. Even if the care episode does not concern the rare disease.
- Writing a systematic review about a rare diseases, or doing a short review in order to write a research article, requires querying one or more bibliographic databases with as much relevant keywords as possible [6].

It seems of public health importance to provide all these participants with the appropriate tools to easily retrieve relevant information about rare diseases.

PubMed is one of the most popular search engines to access medical literature. It browses the MEDLINE bibliographic database, which gathers a large part of biomedical scientific articles, and some other minor resources [7]. MEDLINE is indexed using the MeSH® thesaurus. Although PubMed theoretically allows to access the literature about rare diseases, including the most recent scientific discoveries, the combination of the following elements may hinder users:

- the relative novelty of MeSH terms for rare diseases [8]. Until 2010, the MeSH contained only a few rare diseases, also, citations pertaining to rare diseases published before 2010 are not indexed precisely for rare diseases. Since this date, 10,354 rare diseases, as defined by the Office of Rare Diseases Research (ORDR) [9], have been introduced in MeSH (source MeSH 2014),
- the delay in article MeSH-indexing in PubMed [10], which can be several weeks to several months, according to the importance of the journal, and
- the health professionals, or the lay-persons, lack of knowledge about MeSH [11].

It is therefore difficult for physicians, and furthermore patients, to query Pubmed in an effective way, and especially to find an article about rare diseases published before 2010 or in recent months.

Several institutions (Genetic and Rare Diseases Information Center [12] and Orphanet [13]) already gather information on their website about rare diseases including a brief summary, clinical information and many links to other resources. Sometimes a link to a PubMed expert based query is provided, limiting users task to citation relevance assessment. Nevertheless, in the case of Orphanet these queries do not always take advantage of all the MeSH/PubMed functionalities and they are far from providing a comprehensive coverage of all rare diseases. Moreover, the methodology of establishing these queries is not disclosed on the Orphanet website. The aim of this study was to propose a set of queries linked to each rare disease term in Orphanet and to evaluate these queries against those developed by Orphanet.

## Methods

### PubMed overview

PubMed is the most frequently used bibliographic database used by biomedical scientist throughout the world. It therefore constitute a standard in terms of information retrieval. MEDLINE is the major component of PubMed, gathering almost 90 % of the 26 millions of PubMed citations. MEDLINE curators affect to each citation a list of MeSH terms to describe it with a controlled level of granularity. The MeSH atomic part is the MeSH concept, a class of synonymous terms – i.e. all terms gathered in a MeSH concept are true synonyms. MeSH concepts closely related to each other in meaning may be gathered in a MeSH descriptor (MeSH D) or a MeSH supplementary concept (MeSH SC), one of them being the prefered concept, and the other being narrower, broader or related to the preferred one. Both MeSH D and MeSH SC aims at indexing the citation, but they exhibit some differences. First, MeSH SC are quite specific terms: they are used to index chemicals, drugs, and other concepts such as rare diseases. Second, MeSH SC, unlike MeSH D, are not classed, they are only linked to one or more MeSH D, usually broader, by a specific relationship. Lastly, there are a lot more MeSH SC (≈200,000) than MeSH D (≈27,000).

Pubmed users may specify what search field they want to use in their query using between-bracket operators. Table 1 presents some operators and their meaning.

**Table 1:** Some operators used in PubMed

Operator	Meaning
[ti]	The term is considered as a free text keyword and searched for in title
[ab]	The term is considered as a free text keyword and searched for in abstract
[mh]	The term, a MeSH descriptor, and all the terms it subsumes, are searched for in MeSH indexing
[majr]	The term, a MeSH descriptor, and all the terms it subsumes, are searched for in MeSH major indexing
[nm]	The term, a MeSH supplementary concept, is searched for in MeSH indexing
[tw]	The term is considered as a free text keyword and searched for in multiple fields of PubMed citation (title, abstract, MeSH indexing, other keywords etc.)

### PubMed queries

#### Orpha queries

Orphanet PubMed queries were manually created by Orphanet experts. These queries are available on the Orphanet web site (URL: www.orpha.net), on each disease page (for the diseases that have an Orphanet PubMed query, of course). For exemple, for the Orphanet concept “retroperitoneal fibrosis”, the PubMed query is: retroperitoneal fibrosis[majr] OR Retroperitoneal fibrosis[ti]. For the orphanet concept “Blount disease”, the query is: Blount disease[tw] OR tibia vara[tw].

#### Terminological queries

In addition to the MeSH thesaurus, several other terminologies and ontologies are available on rare diseases: (a) a formal ontology named HRDO (Human Rare Disease Ontology) [14] was developed based on the Orphanet classification. This ontology is available in five European languages: English, French, German, Spanish and Portuguese; (b) the Online Mendelian Inheritance in Man (OMIM) database, developed at Johns Hopkins University [15]; (c) the Human Phenotype Ontology (HPO), a formal ontology, which allows the description in an unambiguous fashion of phenotypic information in medical publications and databases [16]. The HPO is freely available at http://www.human-phenotype-ontology.org.

One of the authors (SJD) has created exact match mappings between MeSH, OMIM, HPO and HRDO based on a natural language processing/conceptual based algorithm [17, 18] suggestions. Exact match mapping means that the two concepts are real synonyms (e.g. the “Absent corpus callosum cataract immunodeficiency” MeSH concept and the “Vici syndrome” HRDO disease). Using these alignments, PubMed queries are created automatically, according to a published algorithm [19]. The algorithm output depends on the type of MeSH term mapped to: MeSH concept, MeSH SC or MeSH D (see Table 2 for examples):

- *If the HRDO concept is mapped to a MeSH Descriptor, the query structure is as follows:*
  Disease[MH] OR Disease[TW] OR Synonyms Disease MeSH Descriptor[TW] OR Synonyms Disease HRDO[TW] OR Synonyms Disease OMIM[TW] (if an exact match mapping exists between HRDO concept and OMIM concept) OR Synonyms Disease HPO[TW] (if an exact match mapping exists between HRDO concept and HPO concept)
- *If the HRDO concept is mapped to a MeSH Supplementary Concept, the query structure is as follows:*
  Disease[NM] OR Disease[TW] OR Synonyms Disease MeSH Supplemntary Concept[TW] OR Synonyms Disease HRDO[TW] OR Synonyms Disease OMIM[TW] OR Synonyms Disease HPO[TW]
- *If the HRDO concept is mapped to a MeSH Concept, the query structure is as follows:*
  Disease[TW] OR Synonyms Disease MeSH Concept[TW] OR Synonyms Disease HRDO[TW] OR Synonyms Disease OMIM[TW] OR Synonyms Disease HPO[TW]
- *And if the HRDO concept is not mapped to the MeSH thesaurus, the query structure is as follows:*
  Disease[TW] OR Synonyms Disease HRDO[TW] OR Synonyms Disease OMIM[TW] OR Synonyms Disease HPO[TW]

**Table 2:** Exemples of queries according to the type of the MeSH term mapped to the HRDO concept

	Types of mapped MeSH terms			
	MeSH descriptor	MeSH supplemetary concept	MeSH concept	Not a MeSH term
HRDO concept example	“retinal dystrophy”	“Omenn syndrome”	“Charcot-Marie-Tooth disease, type ib”	“Isolated oxycephaly”
MeSH part of the query	“retinal dystrophies”[MH] OR “retinal dystrophies”[TW] OR “dystrophies, retinal”[TW] OR “dystrophy, retinal”[TW] OR “retinal dystrophy”[TW] OR	“reticuloendotheliosis, familial, with eosinophilia”[NM] OR “reticuloendotheliosis, familial, with eosinophilia”[TW] OR “severe combined immunodeficiency with hypereosinophilia”[TW] OR	“Charcot-Marie-Tooth disease, type ib”[TW] OR “1B, HMSN”[TW] OR “1Bs, HMSN”[TW] OR	-
HRDO part of the query	“Retinal dystrophy”[TW] OR	“Omenn syndrome”[TW] OR “Combined immunodeficiency with hypereosinophilia”[TW] OR	“Charcot-Marie-Tooth disease type 1B”[TW] OR “CMT1B”[TW] OR	“Isolated oxycephaly”[TW] OR “Turricephaly”[TW] OR “Nonsyndromic oxycephaly”[TW] OR
HPO part of the query	“Retinal dystrophy”[TW]	-	-	“Turricephaly”[TW]
OMIM part of the query	-	“Omenn syndrome”[TW]	“Charcot-marie-tooth disease, demyelinating, type 1b”[TW]	-

Each column contains one example of PubMed query corresponding to the HRDO concept in the “HRDO concept example” row. Each row gathers all the synonyms for the considered diseases in one terminology. The final queries are composed by every synonyms of every terminologies, linked by “OR”. The final PubMed query for the Isolated oxycephalydisease is: “Turricephaly”[TW] OR “Nonsyndromic oxycephaly”[TW] OR “Isolated oxycephaly”[TW]. The last “OR” “turricephaly” is redundant. In this case, the final query is deducible from only one terminology (HRDO)

### Relevance evaluation

Thirty rare diseases were randomly selected from the subset with both an Orphanet query and a terminological query. The selected rare diseases are listed in Table 4 (at the end of the document). The diseases with a prevalence higher than 1/2000 were considered as not rare. One author (GK) gathered the first ten citations retrieved (PubMed “recently added” ranking), for each rare disease, using the following queries:

1 $$ \begin{array}{ccc}\hfill {\mathrm{Q}}_1={\mathrm{Q}}_{\mathrm{Orpha}}\hfill & \hfill \mathrm{AND}\hfill & \hfill {\mathrm{Q}}_{\mathrm{Term}}\hfill \end{array} $$

2 $$ \begin{array}{ccc}\hfill {\mathrm{Q}}_2={\mathrm{Q}}_{\mathrm{Orpha}}\hfill & \hfill \mathrm{NOT}\hfill & \hfill {\mathrm{Q}}_{\mathrm{Term}}\hfill \end{array} $$

3 $$ \begin{array}{ccc}\hfill {\mathrm{Q}}_3={\mathrm{Q}}_{\mathrm{Term}}\hfill & \hfill \mathrm{NOT}\hfill & \hfill {\mathrm{Q}}_{\mathrm{Orpha}}\hfill \end{array} $$

With Q_Orpha_ the Orpha query and Q_Term_ the terminological query. Therefore, Q_1_ retrieved citations common to both Orpha and terminological query, Q_2_ retrieved citations specific to the Orpha query and Q_3_ retrieved citations specific to the terminological query. He (GK) then hid the retrieving query: the evaluators were blinded vs. the type of query. The anonymised citations were split between four physicians (FD, LR, MS and NG) in such way that: (i) each citation was evaluated twice and, (ii) each evaluator shared each third of their evaluations with one different evaluator.

Evaluators had to answer the following question for each citation: “Does the article directly concern the disease?” In case of any disagreement, a third evaluator evaluated the citation and the discrepancy was resolved by consensus.

More information regarding relevance evaluation is available in Additional file 1.

### Statistical analysis

Agreement between evaluators was measured by kappa. HRDO rare diseases may be split into two: terms with an Orpha query and terms without Orpha query. These two sub-populations were compared according to available determinants to ensure generalizability.

For each rare disease, it is possible to estimate the precision (p_i_) of each query (Q_1_, Q_2_, Q_3_; see Eq. 4).

4 $$ {\mathrm{p}}_{\mathrm{i}}=\mathrm{n}\left({\mathrm{rel}}_{\mathrm{Qi}}\right)/\mathrm{n}\left({\mathrm{eval}}_{\mathrm{Qi}}\right) $$

With n(rel_Qi_) and n(eval_Qi_) the number of relevant citation and the number of evaluated citation for the query i, respectively. Orpha queries and terminolgical queries were compared according to micro average precision, number and publication date of retrieved citations, and use of MeSH terms. Non-parametric tests were used: Fisher’s test for qualitative variables (micro average precision and MeSH use) and Wilcoxon test and Kruskal-Wallis test for quantitative variables (number of citation and date). The Dunn test allows pairwise comparison after Kruskal-Wallis.

## Results

HRDO, in its 09/11/2013 version, inventory 9060 diseases and groups of diseases. Seventy-eight were not considered as rare diseases because the prevalence, as specified by Orphanet, was above the European threshold, also, the study considered only the 8982 rare diseases. Table 3 lists the number of alignments created or validated by SJD.

**Table 3:** Number of exact match mappings created between the different terminologies considered

	MeSH			HPO	OMIM
	Descriptor	Supplementary Concept	Concept		
HRDO	1247	2620	3837	484	2707
OMIM	550	4019	4681	296	
HPO	886	157	1131		

For example, SJD has created 1247 synonymy mappings between an HRDO concept and a MeSH descriptor

**Fig. 1:**
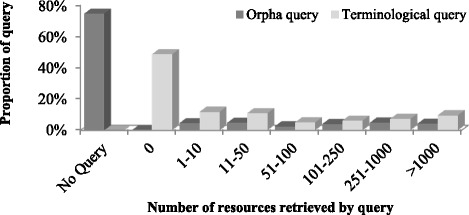
Distribution of queries according to the number of citations retrieved for Orphanet and terminological queries

Only 2284 HRDO rare diseases have a manually validated Orphanet query (25.4 %). A terminological query is generated for each disease in Orphanet (was it rare or not). Orpha queries and terminological queries respectively retrieved 0 citations in 5 (<1 %) and 4370 (48.7 %) cases (see Fig. 1). Considering both “no query” and “0 citations” situations, there is a useful terminological or Orpha queries for 51.3 or 25.4 % of HRDO rare diseases, respectively.

The 30 selected rare diseases and the number of citations retrieved by each query are listed in Table 4. Terminological queries retrieved more citations more often than Orpha queries (17 terminological queries retrieved more results than Orpha queries while only 10 orpha queries retrieved more results than terminological queries; *p* = 0.01; Wilcoxon test). As some queries retrieved less than 10 citations (see Table 4), only 553 PubMed citations were assessed for relevance (instead of 30 × 3 × 10 = 900). Kappa indexes before the adjudication procedure range from moderate (0.41) to almost perfect (0.86) agreement. Overall kappa was 0.68 (substantial agreement).

**Table 4:** Number of citations retrieved and precision for each query, by diseases

Disease	MeSH level alignment	n(retr)			Precision		
		Q_1_	Q_2_	Q_3_	p_1_	p_2_	p_3_
3M syndrome	SC	38	5	31	1	0.4	0.4
Autosomal recessive hypohidrotic ectodermal dysplasia	D	25	21	12	0.8	0.4	0.8
Generalized epilepsy - paroxysmal dyskinesia	SC	0	18	6	–	0.5	0.17
Silent sinus syndrome	–	107	2	3	1	1	0.67
Toluene embryopathy	SC	8	39	0	1	0.2	–
Familial drusen	SC	67	54	9	1	0.1	0.78
Autoimmune lymphoproliferative syndrome	D	176	5	1800	1	1	0
Diphtheria	D	4419	0	13,149	1	–	0.6
Hypomandibular faciocranial dysostosis	SC	6	2	0	0.83	1	–
Retroperitoneal fibrosis	D	1986	0	711	1	–	0.6
Epstein syndrome	SC	41	0	0	1	–	–
Oculopharyngeal muscular dystrophy	D	291	31	129	0.9	1	0.6
Ring chromosome 19	SC	6	4	2	1	0.75	1
Nephropathy - deafness - hyperparathyroidism	SC	0	1	0	–	1	–
Greenberg dysplasia	SC	7	4	6	1	1	0.83
Menkes disease	D	968	0	47,026	1	–	0
Mikati-Najjar-Sahli syndrome	–	0	1	0	–	1	–
Genochondromatosis	SC	5	0	0	1	–	–
Noonan syndrome	D	1483	0	258	0.8	–	0.5
Carney complex	D	248	21	299	0.9	1	0.7
Blount disease	SC	296	0	6	0.5	–	1
Oculocerebrofacial syndrome, Kaufman type	SC	5	0	0	1	–	–
Wilson disease	D	5266	0	1231	1	–	0.8
Adult Still’s disease	D	1129	0	202	1	–	0.8
Esophageal atresia	D	2999	75	479	0.9	1	0.6
Congenital nephrotic syndrome, Finnish type	C	8	239	43	0.88	0.8	0.1
Thiamine-responsive megaloblastic anemia syndrome	SC	69	34	39	1	0.6	0.1
Hereditary myoclonus - progressive distal muscular atrophy	SC	0	2	0	–	0.5	–
Dentatorubral-pallidoluysian atrophy	C	361	171	215	0.9	0	0.6
Neuronal ceroid lipofuscinosis	D	1371	0	1379	1	–	0.5
Macro average precision					0.81	0.44	0.41
Micro average precision					0.94	0.61	0.52

*n(retr)* number of citations retrieved, *D* MeSH Descriptor, *SC* MeSH Supplementary Concept, *C* MeSH Concept

The precision of each query, computed after adjudication process, are listed in Table 4. The intersection query (Q_1_) is significantly more precise than Q_2_ (*p* = 0.01; Fisher’s test) and Q_3_ (*p* < 0.001; Fisher’s test). However, there was no significant difference between Q_2_ and Q_3_ precision (0.61 vs. 0.52, respectively; *p* = 0.13; Fisher test). For the 30 selected diseases, there was significantly more terminological query that fully used the MeSH thesaurus (28 vs. 8; *p* < 0.001; Fisher’s test; data not shown).

When considering relevant citations alone, it is noteworthy that citations retrieved by Q_2_ (i.e. only by orpha queries) are significantly older than those retrieved by Q_1_ and Q_3_ (*p* < 0.0001 in both cases, Dunn’s test with Bonferroni correction). Median publication dates are 2013, 2005 and 2014 for Q_1_, Q_2_ and Q_3_, respectively. The results are very similar when considering all the citations evaluated, whether they were relevant or not.

## Discussion

There is no differences between Orpha and terminological queries in terms of precision. However, Orpha queries retrieved significantly fewer results. Moreover, citations retrieved only by Orpha queries are significantly older than citations retrieved by terminological queries, and, Orphanet provides queries for only 25.4 % of rare diseases while terminological queries retrieved at least one PubMed citation for 51.3 % rare diseases. This suggests a differentiated approach according to the user objectives:

- a precision-interested user should use the intersection query, which will retrieve the most relevant citations,
- a recall-interested user might be interested in the union query.

Nevertheless, for almost 75 % of HRDO rare disease, there is no other solution but the terminological query.

Physicians are probably more interested in precision than in recall. A researcher, in contrast, may be more interested in recall for their literature review. However, in many cases, only the terminological query is available leaving the user no choice. A potentially interesting use of the set of terminological queries is its use to find medical experts about rare diseases [5], where noise is a less important problem. The set of terminological queries is available from the Health Terminology Ontology Portal [20] (URL: http://www.hetop.eu).

Two mechanisms may explain the more up to date set of results retrieved by terminological query: (i) the major part of the difference is a consequence of the evaluation method. As terminological queries retrieve more results than orphanet queries, we can hypothesized that there is both more recent and more older citations. However, PubMed ranks recent results first and we only evaluated the first ten results – i.e. the more recent. (ii) Some keywords added by the terminology expansion are quite recent, and not yet taken into account by orphanet expert in their queries.

While these hypotheses limit the value of the up-to-date effect of terminological query, it raises a maintenance issue. Creating and maintaining a query is very time-consuming and it is probably one of the main limitation of Orphanet query. For terminological queries, the maintenance may only be necessary when terminologies evolve. For example, Vasilevsky et al. [21] recently enhanced HPO with terms that patients, doctors, and machines can all understand. This evolution will require a limited validation maintenance for terminological queries. However, the convergence of these terminologies (with the Orphanet Rare Diseases Ontology [22]) may ultimately importantly reduce the maintenance tasks.

### Strengths and limitations

Only two sets of queries were compared in this study: one from the Orphanet [13] and one based on terminologies [20]. The queries from the Genetic and Rare Diseases Information Center [12] were not tested for this study because of their limited design (they often only rely on OMIM record references [23], which are not updated on a regular basis). In fact, these queries cannot retrieve any citation that has not been considered by the OMIM authors. The added-value against the OMIM record references is therefore very limited. Also, only two set of queries may be considered as gold standard for a terminology queriescomparison: Orpha query and free text queries, which most users are likely to submit. They both present pros and cons.

Using free text query sounds like a very pragmatic approach, close to the reality. Results would be easy to interpret. Nevertheless, it is difficult to establish due to the impossibility of formalizing such a gold standard. The label choice has a major influence over the query result: if a label from the MeSH is used, PubMed will automatically recognize the MeSH term and perform a semantic expansion, otherwise, the query may be tokenized and each term searched for separately, which would introduce a lot of noise.

Using Orpha query may seem questionnable: only 25.4 % of the rare disesases are provided with a query and query production processus is unclear. However, these queries are somewhat validated by Orphanet expert and they are available online.

For these reasons, the use of Orpha query as a gold standard seemed to be preferable. The question the evaluators have to answer for each citation is quite generic and it might not be adapted to the real users context. One difficulty is to reach an acceptable inter evaluator agreement, the only way to assess the quality of the relevance assessment. A more specific question was tested: “Is the citation useful for medical care?” but agreement was very low.

The main limitation of this study is probably the quality assurance of terminology mapping: relying on one expertise is not sufficient for sensitive data, and while the help of an automatic algorithm may limit the false positive rate at the same time it also increases the false negative rate. Also, proper quality assurance might probably have slightly enhance terminological query performance. Nevertheless, the results presented in our study, with no difference in precision, demonstrate that a sufficient high mapping quality was achieved.

This study demonstrates some strengths. First, the evaluation of citations by two independant physicians unaware of the query and the adjudication procedure render the judgement as reliable and unbiased as possible. Second, the results are theoretically generalizable because of the random selection of the diseases, which led to a similar distribution of disease prevalence in the studied corpus compared to the entire HRDO.

The main strength of the terminological approach presented here is the availability of a query for each rare disease in each terminology. The cost of this approach – maintenance of mapping – seems very limited. Queries take advantage of the rich synonymy of classifications (HPO, HRDO, OMIM, MeSH), and, when there is an alignment to MeSH, of MeSH indexing. The semantic expansion used here could be enhanced using UMLS, nevertheless, this resource has already been shown to be too noisy [24].

### Query structure - MeSH

Orpha queries and terminological queries are structurally different. Terminological queries are based on the automatic exploitation of terminological knowledge, therefore the queries are structurally simple, i.e. all the keywords are linked by a “OR” in the query. Orpha query, as manually designed, may be more complex, implying all the boolean operators (AND, OR and NOT). Even if an exact match MeSH term does not exist it is possible to use a combination of MeSH terms relevant to the disease. Overall, as previously mentioned above, the creation and maintenance of Orpha queries is a much more time consuming task.

MeSH use is also problematic because of the novelty of rare disease MeSH terms [8]. Therefore, decades of citations about rare diseases are only indexed using free text and MeSH term recall is necessarily low. Nevertheless, the indexing of citations with MeSH will gradually increase, enhancing the recall of queries based on MeSH terms, the mapping between Orphanet diseases and MeSH terms is therefore important to maintain.

## Conclusions

There is a terminological query for each rare disease. This query precision was not statistically different from the precision found for Orpha queries. The terminological queries proposed in this study are a useful tool for both precision or recall oriented literature search in combination with the Orpha query, if available.

## Abbreviations

MeSH, medical subject headings; MeSH D, MeSH descriptor; MeSH SC, MeSH supplementary concept; OMIM, Online Mendelian Inheritance in Man; ORDR, Office of Rare Diseases Research; HPO, human phenotype ontology; HRDO, human rare disease ontology; USA, United States of America

## Additional file

Additional file 1: Contains all the evaluated citation, their metadata and their final evaluation. As authors are French, this file is in French, however, the entire work was performed in English. Column 1 contains the unique disease ID, column 2 the disease name, column 3 the query, column 4 the MeSH level, column 5 the citation PubMed ID, column 6 the final answer for relevance (“Does the article directly concern the disease?”), column 7 the year of publication, column 8 the journal title, column 9 a link toward the citation, column 10 and 11 the two evaluators, column 12 the adjudicator, if any. (XLSX 62 kb)

## References

[1] Field MJ, Boat TF, Institute of Medicine (US) Committee on Accelerating Rare Diseases Research and Orphan Product Development (2010). Profile of Rare Diseases. Rare Dis Orphan Prod Accel Res Dev.

[2] U.S. and World Population Clock. http://www.census.gov/popclock/. Accessed 13 Jul 2016.

[3] Official Journal of the European Union C151, 3.7.2009, p 7

[4] Forman J, Taruscio D, Llera VA, Barrera LA, Coté TR, Edfjäll C (2012). The need for worldwide policy and action plans for rare diseases. Acta Paediatr.

[5] Pflugrad A, Jurkat-Rott K, Lehmann-Horn F, Bernauer J (2014). Towards the Automated Generation of Expert Profiles for Rare Diseases through Bibliometric Analysis. Stud Health Technol Inform.

[6] Higgins JPT, Green S (2011). Cochrane handbook for systematic reviews of interventions Version 5.1.0 [updated March 2011]. The Cochrane Collaboration.

[7] MEDLINE, PubMed, and PMC (PubMed Central): How are they different?. http://www.nlm.nih.gov/pubs/factsheets/dif_med_pub.html. Accessed 13 Jul 2016.

[8] Schulman JL (2010). What’s New for 2011 MeSH®. NLM Tech Bull.

[9] Office of rare diseases research. http://rarediseases.info.nih.gov. Accessed 13 Jul 2016.

[10] Huang M, Névéol A, Lu Z (2011). Recommending MeSH terms for annotating biomedical articles. J Am Med Inform Assoc.

[11] Hoogendam A, Stalenhoef AF, Robbé PF, Overbeke AJ (2008). Analysis of queries sent to PubMed at the point of care: observation of search behaviour in a medical teaching hospital. BMC Med Inform Decis Mak.

[12] Genetic and Rare Diseases Information Center. http://rarediseases.info.nih.gov/gard. Accessed 13 Jul 2016.

[13] Orphanet. http://www.orpha.net/consor/cgi-bin/index.php. Accessed 13 Jul 2016.

[14] Dhombres F, Vandenbussche P-Y, Rath A, Olry A, Hanauer M, Urbero B, et al. OntoOrpha: an ontology to support edition and audit of rare diseases knowledge in Orphanet. Proceedings of the 2nd International Conference on Biomedical Ontology (ICBO-2011). Buffalo, NY, USA: Olivier Bodenreider, Maryann E. Martone, Alan Ruttenberg (eds.); 2011, p 241–3

[15] Amberger J, Bocchini CA, Scott AF, Hamosh A (2009). McKusick’s online mendelian inheritance in man (OMIM). Nucleic Acids Res.

[16] Robinson PN, Mundlos S (2010). The Human Phenotype Ontology. Clin Genet.

[17] Merabti T, Soualmia LF, Grosjean J, Palombi O, Müller JM, Darmoni SJ (2011). Translating the Foundational Model of Anatomy into French using knowledge-based and lexical methods. BMC Med Inform Decis Mak.

[18] Merabti T, Joubert M, Lecroq T, Rath A, Darmoni S (2010). Mapping biomedical terminologies using natural language processing tools and UMLS: mapping the Orphanet thesaurus to the MeSH. Biomedical Engineering and Research.

[19] Thirion B, Robu I, Darmoni SJ (2009). Optimization of the PubMed Automatic Term Mapping. Stud Health Technol Inform.

[20] Grosjean J, Merabti T, Griffon N, Dahamna B, Darmoni SJ (2012). Teaching medicine with a terminology/ontology portal. Stud Health Technol Inform.

[21] Vasilevsky N, Engelstad M, Foster E, McMurry J, Mungall C, Robinson P, et al. Finally, a medical terminology that patients, doctors, and machines can all understand. http://human-phenotype-ontology.github.io/2016/03/24/layperson.html. Accessed 13 Jul 2016.

[22] Vasan D, Chanas L, Malone J, Hanauer M, Olry A, Jupp S (2014). ORDO: An Ontology Connecting Rare isease, Epidemiology and Genetic Data. Proc. PhenoDay and Bio-Ontologies at ISMB.

[23] Online Mendelian Inheritance in Man®. http://www.omim.org/. Accessed 13 Jul 2016.

[24] Griffon N, Chebil W, Rollin L, Kerdelhue G, Thirion B, Gehanno JF (2012). Performance evaluation of Unified Medical Language System®’s synonyms expansion to query PubMed. BMC Med Inform Decis Mak.

